# An Adrift and Homeless Cyst within the Peritoneal Cavity

**DOI:** 10.1155/2021/6630043

**Published:** 2021-12-23

**Authors:** Ali Mehri, Aida Ayati afin, Masoumeh Gharib, Mohammad Etezadpour

**Affiliations:** ^1^Student Research Committee (SRC), Faculty of Medicine, Mashhad University of Medical Sciences, Iran; ^2^Department of Pathology, Faculty of Medicine, Mashhad University of Medical Sciences, Mashhad, Iran; ^3^Department of General Surgery, Faculty of Medicine, Mashhad University of Medical Sciences, Mashhad, Iran

## Abstract

**Introduction:**

Echinococcosis is a zoonotic infection caused by *Echinococcus* species. Iran is endemic for *Echinococcus granulosus*. Here, we present a case of hydatidosis in an 85-year-old man, presented with acute, dull, constant, and generalized abdominal pain. A computed tomography scan (CT scan) showed an intact hydatid cyst on the bladder dome and several hydatid cysts in the liver. Open surgery revealed a cyst with hepatic origin, confirmed with histopathological studies.

**Conclusion:**

Although a primary abdominal hydatid cyst is very rare, it can be transferred to the abdominal cavity without any rupture as secondary ones. In this case, rupture of the liver wall was the reason for this transfer. As a result, there should be a suspicion of hydatidosis in a patient with a similar presentation.

## 1. Introduction

Echinococcosis is a zoonotic infection caused by a parasitic tapeworm called *Echinococcus* from the *Taeniidae* family. Echinococcosis, based on its transmission cycle, has specific geographical distribution around the world. The Middle East, especially Iran, is among the endemic places for *Echinococcus granulosus* [1]. According to a 2015 report by the World Health Organization, it costs more than $3 billion, including treating patients and causing damage to the livestock industry every year. The report also states that the disease causes 19,300 deaths and around 871,000 disability-adjusted life-years (DALYs) every year, all around the world [2].

Here, we discuss a rare presentation of hydatid cyst and the purpose of this report is to draw the attention of physicians to include hydatid cyst in the differential diagnosis of patients with similar manifestations.

## 2. Case Presentation

An 85-year-old healthy man presented to the surgery clinic of Ghaem Hospital, complaining of abdominal pain, which was generalized, dull, and acute and started two days before he came to the clinic. The pain was constant, and he declared its intensity 4 out of 10, with no radiation. Moreover, he did not reveal any correlation between pain intensity and eating or activity. He did not mention any recent fever, nausea, or vomiting. He did not report any past medical history of surgery or hospitalization.

On his medical examination, vital signs were as follows: heart rate = 98/min, respiratory rate = 14/min, blood pressure = 115/70 mmHg, and axial temperature = 37.3°C. His abdomen was soft, and there was just a mild generalized tenderness without rebound or guarding. There was no sign of organomegaly, scar, or herniation. The rest of his examination was normal.

Complete blood count was normal, and blood chemistry did not show any abnormal findings other than high ESR (53 mm/hr) and high CRP (187 mg/l) with the normal range of ESR (0-22 mm/hr for men) and CRP < 10 mg/l.

**Figure 1:**
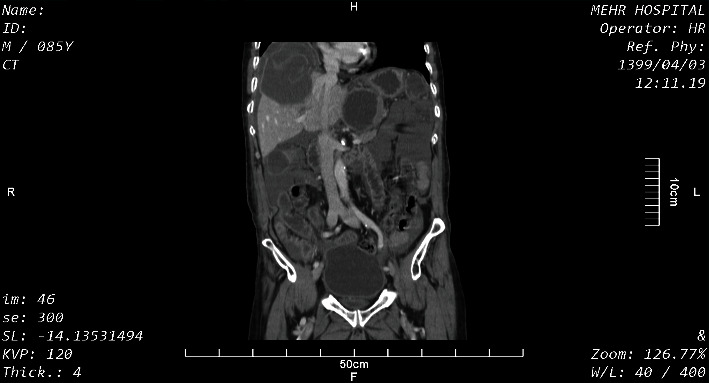
A coronal section of the abdomen and pelvis with IV contrast.

**Figure 2:**
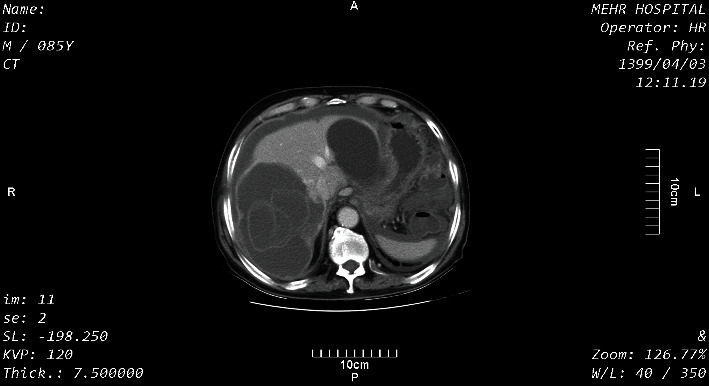
An axial section of the abdomen with IV contrast.

**Figure 3:**
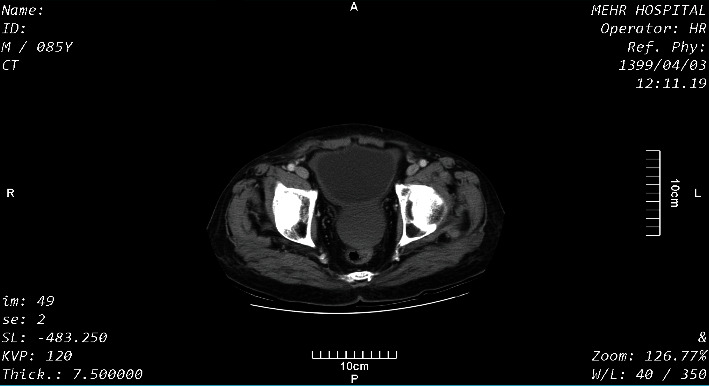
An axial section of the pelvis with IV contrast.

Coronal sections of the abdomen-pelvic CT scan with IV contrast showed a hepatic hydatid cyst with water lily sign in the right lobe of the liver with a compressive effect on the adjacent diaphragm and the left lobe of the liver and hydatid cyst of the bladder dome (Figure 1). In axial sections of the CT scan, hepatic hydatid cysts were seen with water lily sign and pressure effect on the diaphragm and an intact capsule in the right and the left lobes of the liver. Free abdominal fluid was present (Figure 2). In other axial sections of the pelvis, a hydatid cyst was seen with an intact capsule on the bladder dome (Figure 3).

Open abdominal surgery was performed with the final diagnosis of hydatidosis. There was an intact cyst without any invasion right above the bladder with 10∗13 cm size and hepatic origin. There was no adhesion, but just a little fibrous tissue was seen between the cyst and bladder.

**Figure 4:**
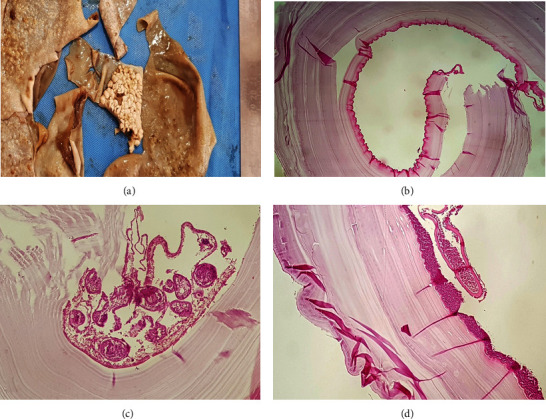
Hydatid cyst. (a) Gross appearance of the cyst, which shows multiple foci of fibrin deposition and daughter cysts. (b) Laminated, hyaline wall of hydatid cyst, lined by germinative layer. (c) A daughter cyst, containing multiple scolices (a brood capsule). (d) Collections of neutrophils, covering the outer surface of hydatid cyst.

Further examination of the liver revealed a perforation, and the source of intra-abdominal bloody discharge was from there. Isolated hepatic cysts were aspirated and washed with hypertonic serum. The cysts were not related to the bile ducts, and bile secretions were not seen at the site of the cyst cavity. Pathology samples were sent for further examination. On histopathological examination, hydatid cyst diagnosis was confirmed for the bladder dome cyst (Figure 4).

The postoperative status was good and stable. The patient was discharged four days after the surgery with an albendazole prescription (10 mg/kg, twice a day) and said to visit the surgical clinic next week.

## 3. Discussion

Hydatid cyst occurs due to the cystic form of *Echinococcus* spp. Echinococcosis is endemic in Iran with a prevalence of 5% and an estimated expense of US$93.39 million. The disease is more frequent in southwest and south of Iran mostly in rural regions and is accountable for 1% of the admissions to surgical wards [3].

CE is considered to be endemic in all eastern Mediterranean region, and this region is one the major hotspots of CE. Despite this fact, CE is not among the health topic list of diseases in this region which requires more attention [4]. In Pakistan as one of these countries, CE is also endemic but their data is still limited [5].

Moreover, in India which is not far from Iran, the incidence varies from 1 to 200/100,000 in a year and most of the cases were from Kashmir, Andhra Pradesh, Tamil Nadu, and Central India [6].

Most cases are asymptomatic for decades and are found accidentally or in the autopsies. However, there are several clinical signs and symptoms for cystic echinococcosis, which is different based on the size and involved organ. The cyst size is wavering, but often it is between 1 and 15 centimeters [7, 8].

Different organs are involved in this disease, and the liver and lungs are the most common sites, respectively.

Most symptoms of cystic echinococcosis (CE) are due to the gradual growth of the cyst, which puts pressure on the adjacent structures, which could cause abdominal discomfort, pain, nausea, and vomiting. The rupture of CE that can be caused by blunt or spontaneous trauma is an acute complication. The ruptures are of three different types, and based on the type, there could be no leakage or leakage to the biliary system or the abdominal cavity [9, 10].

In this case, the CE was intact, mobile in the peritoneal cavity right above the bladder with hepatic origin, and the rupture happened in the liver wall. There were multiple foci of fibrin deposition on CE and no adhesion to the bladder. Just Kahraman et al. have reported a similar wandering cyst in the peritoneal cavity. However, it was a daughter cyst caused by a ruptured primary cyst in the liver [11]. Therefore, it is the first report of an intact and unruptured CE in the abdomen. In general, primary pelvic hydatid cysts are very rare [12], but they can induce urinary disorders and menstruation irregularity [13].

## 4. Conclusion

In conclusion, a hydatid cyst should be considered as one of the differential diagnoses of acute abdominal pain. Although the primary abdominal hydatid cyst is very rare, it can transfer to the abdominal space without any rupture as a secondary abdominal hydatid cyst. Therefore, it is crucial to look for other hydatid cysts in other organs, such as the liver.
